# Are Orienteers Protected Enough against Tick Bites? Estimating Human Exposure to Tick Bites through a Participative Science Survey during an Orienteering Competition

**DOI:** 10.3390/ijerph18063161

**Published:** 2021-03-18

**Authors:** Jonas Durand, Laure Bournez, Julien Marchand, Claire Schmid, Irene Carravieri, Béatrice Palin, Cyril Galley, Vincent Godard, Annick Brun-Jacob, Jean-François Cosson, Pascale Frey-Klett

**Affiliations:** 1 Tous Chercheurs Laboratory, UMR 1136 ‘Interactions Arbres Micro-Organismes’, INRAE—Lorraine University, Centre INRAE Grand Est-Nancy, F-54280 Champenoux, France; irene.carravieri@citique.fr (I.C.); beatrice.palin@inrae.fr (B.P.); annick.brun@univ-lorraine.fr (A.B.-J.); 2 Nancy Laboratory for Rabies and Wildlife, The French Agency for Food, Environmental and Occupational Health and Safety (ANSES), F-54220 Malzéville, France; cjuliette.schmid@gmail.com; 3 CPIE Champenoux, F-54280 Champenoux, France; julien.marchand.cpie54@gmail.com (J.M.); cyril.galley@cpie54.com (C.G.); 4 Department of Geography, Université Paris 8, UMR LADYSS CNRS, F-93200 Saint-Denis, France; vgodard@univ-paris8.fr; 5 UMR BIPAR, INRAE, F-94700 Maisons-Alfort, France; 6 US 1371 Laboratory of Excellence ARBRE, INRAE, Centre INRAE Grand Est-Nancy, F-54280 Champenoux, France; pascale.frey-klett@inrae.fr

**Keywords:** tick, citizen science, tick-bite exposure, prevention, *Ixodes ricinus*

## Abstract

Mass-participation events in temperate forests are now well-established features of outdoor activities and represent high-risk activities regarding human exposition to tick bites. In this study we used a citizen science approach to quantify the space–time frequency of tick bites and undetected tick bites among orienteers that participated in a 6-day orienteering competition that took place in July 2018 in the forests of Eastern France, and we looked at the use and efficacy of different preventive behaviors. Our study confirms that orienteers are a high-risk population for tick bites, with 62.4% of orienteers bitten at least once during the competition, and 2.4 to 12.1 orienteers per 100 orienteers were bitten by ticks when walking 1 km. In addition, 16.7% of orienteers bitten by ticks had engorged ticks, meaning that they did not detect and remove their ticks immediately after the run. Further, only 8.5% of orienteers systematically used a repellent, and the use of repellent only partially reduced the probability of being bitten by ticks. These results represent the first attempt to quantify the risk of not immediately detecting a tick bite and provide rare quantitative data on the frequency of tick bites for orienteers according to walking distance and time spent in the forest. The results also provide information on the use of repellent, which will be very helpful for modeling risk assessment. The study also shows that prevention should be increased for orienteers in France.

## 1. Introduction

In Europe, ticks are the most important vectors of pathogens with medical or veterinary importance [1]. In the past decades, the incidence of tick-borne diseases such as Lyme disease has increased throughout Europe [2,3,4] and can be linked to an increase in tick bites [5]. The most common tick species biting humans in western Europe is the hard tick *Ixodes ricinus* [6,7]. *Ixodes* ticks have three developmental stages. The two immature tick stages, larva and nymph, take a single blood meal to develop into the next stage, and only the female adult has to feed once again in order to lay eggs while the male does not need to feed. The three stages live in the vegetation and feed on different hosts, which can be as different as mammals, birds or reptiles [7], but humans are mostly bitten by nymphs, followed by larvae [8,9,10,11,12,13]. *Ixodes ricinus* ticks can be the vector of different human pathogens: bacteria such as *Borrelia burgdorferi* sensu lato, an agent of Lyme disease, parasites such as *Babesia divergens*, or viruses such as the tick-borne encephalitis virus [7,14]. Transovarial transmission is absent or rare for the most common bacterial pathogen, *B. burgdorferi* sl [15,16,17], so larval stages are considered less dangerous for human health than other stages even if they are very small (less than 1 mm) and consequently more difficult to detect. The risk of transmission of a tick-borne bacterial pathogen increases with the duration of attachment of the tick on humans. Indeed, an infected tick generally needs 12–24 h to transmit a bacterium, e.g., *Borrelia burgdorferi* sl., and few minutes or hours to transmit a virus [18]. A vaccine exists against tick-borne encephalitis, but for other tick-borne diseases transmitted by *Ixodes ricinus* ticks, the best prevention method relies in preventing tick bites, or at least encouraging tick checks after a risky activity [14,19]. Searching for attached ticks is a good way to reduce the duration of the tick blood meal and thus the risk of transmission of a bacterial pathogen [20,21]. Preventing tick bites usually prevents tick contact [14,22,23,24]. This can be done by using protective clothes, chemical repellents, or by avoiding highly infested tick areas.

The risk for people being in contact with a tick-borne pathogen is not only defined by (i) the density of infected ticks in an environment people are visiting (i.e., the fundamental risk of encountering ticks) but also (ii) by the likelihood that people engage in activities in tick-infested areas and are in contact and bitten by at least one infected tick attached for long enough to transmit a pathogen. This second component depends on how people use the landscape and behave (frequency, type of activity, space–time exposure) and also protect themselves [4,25,26,27,28]. Therefore, the likelihood of people engaging in activities in tick-infested areas and being in contact with and bitten by at least one infected tick is one of the most difficult parameters to quantify when assessing the risk of contact of tick-borne pathogens [25], and so far, very few studies have attempted to quantify this [29]. The likelihood depends partly on the space–time exposure of people to tick-infested areas, i.e., the overall time they spend and the distance they travel in these areas, as well as on the type of activity they conduct in tick-infested areas (hiking, running, mushroom picking).

In Europe, environments with a high density of ticks consist mostly of forests [30,31]. In the past decades, there has been an increase in outdoor recreational activities in forests [32,33] that may have resulted in a higher exposure to tick-borne diseases [28]. Some activities are particularly at risk, such as scouting [34], marathons [29] or orienteering [35,36,37,38,39]. However, quantitative data on the space–time exposure risk of tick bites, on the risk of not detecting a biting tick, and on the preventive measures that are used by people during these activities are still sparse, especially in France. Such data are crucial for public health police makers to adapt their prevention campaign and are also important input for modelling human risk exposure and risk assessment. Mass-participation events, ideally suited to engage citizens in science [25,29], provide a unique opportunity to obtain such quantitative data by providing a large sample of data on people in the same environment at the same time. Using a citizen science approach can not only provide a large sample of data that would have been difficult to collect, but this is also a good way to improve tick-borne disease prevention among contributors [40,41,42].

In France, the citizen science program CiTIQUE (www.citique.fr, accessed on 16 March 2021) was created in 2017 to facilitate collaboration between citizen and scientists on tick and tick-borne pathogen issues. Its main purpose is to study the ecology of ticks and tick-borne diseases in order to improve prevention. As part of this program, a small research project was organized to study two components of the risk of tick-borne pathogen transmission to humans during a 6-day orienteering international competition: (i) the space–time exposure to tick bites, which includes prevention methods that people use and (ii) the risk of being bitten by a tick for long enough to transmit pathogens if the tick is infected (i.e., the risk of not detecting a tick bite). Space–time exposure of orienteers to tick bites during this race was assessed by identifying the ticks biting orienteers and studying the frequency of tick bites per day according to the overall distance and time orienteers spent in the forest. To consider the contribution of prevention methods to decrease the global risk of being bitten, we studied (i) the use of tick repellents among orienteers and how effective they were to prevent tick bites and (ii) the frequency of undetected tick bites, while heavily promoting post-run tick checks during the competition, represented by the number of engorged ticks collected.

## 2. Materials and Methods

### 2.1. Sample Collection

**Figure 1:**
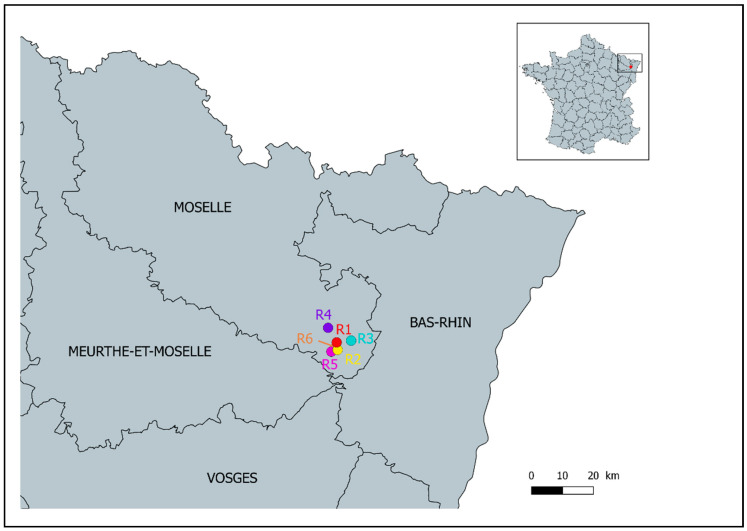
**Localization of the orienteering competition.** R1 to R6 correspond to racing rounds 1 to 6. R6 took place very close to R5 and cannot be displayed on the map.

An international orienteering competition was held from the 8th to the 14th of July 2018 in forests of Moselle, East France (Figure 1). The event comprised six rounds of racing during six days: the first day was a “sprint race” (1–2 km); the 2nd, 4th, and 6th days were long-distance racing rounds (3–14 km); the 3rd and 5th days were medium-distance racing rounds (2–6 km). Overall, 1491 persons aged from 8 to 85 years old participated in this competition. Participants were from 23 countries, mostly from France (71%) and Belgium (9%). The race length and course varied according to race categories, defined by age and gender. The racing rounds took place between 9:00 am and 3:00 pm, with the starting time ranging from 9:00 am to 12:20 pm.

A stand was held by volunteers of the CiTIQUE citizen science program to collect information on tick bites during this international orienteering race. A giant banner was displayed at this stand, on which volunteer orienteers were asked to write at the end of each racing round whether or not they were bitten by ticks and if they used a repellent. On the banner, orienteers were identified by their bib number. From the second day of the competition, orienteers were also asked to place any ticks they found biting their bodies after the race into a tube containing 70% ethanol and to fulfill the associated form with the following information: bib number, date, and use of repellent. CiTIQUE volunteers also offered a tick-removal service and gave information on ticks and tick-borne diseases at the stand, which served to encourage wider participation. Before and during the event, the objectives and methods of our study were communicated to all competitors via the O’France website (http://www.o-france.fr/2018-foot/informations/stand-balance_tes_tiques/, accessed on 17 December 2020), the competition program leaflet, and by oral announcements during the competition.

**Figure 2:**
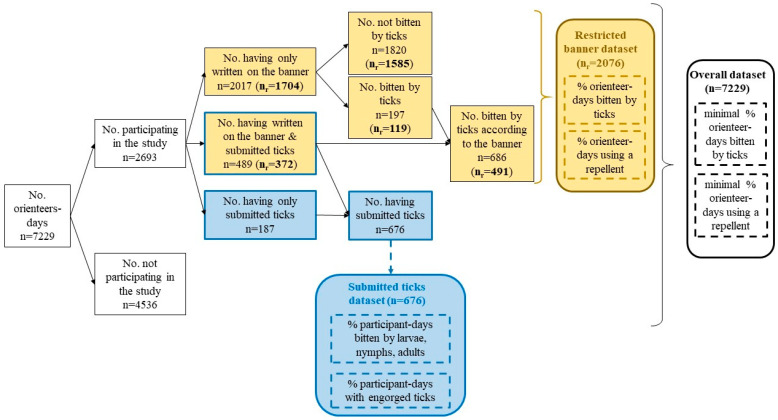
**Description of the different groups that were created from the orienteers for the different analysis.** An orienteer-day refers to one orienteer during one day, without considering if the orienteer participated in the study on another day. We differentiated data from the giant banner (in yellow) and data from the biting ticks submitted at the end of each racing round (in blue). Dotted lines indicate parameters estimated from our analysis; straight lines indicate parameters directly measured. n_r_ refers to the banner-restricted dataset: orienteers who participated at least four times in the study.

Different subgroups were identified in the sample: people who wrote on the banner and submitted biting ticks, people who only wrote on the banner, and people who only submitted ticks without giving information on the banner. Each group could be analyzed separately or together, depending on the type of analysis used. In order to facilitate the understanding of further analysis, we summarized the different groups in Figure 2. Hereinafter, we used the term “banner study” when people wrote the information on the banner. 

We recorded the developmental stages of ticks and identified the species morphologically in the laboratory. We identified the ticks that displayed any morphological changes associated with feeding and considered them as “engorged”. Since morphological changes associated with feeding are visually detectable for ticks feeding for more than 10 h [43], we considered that these “engorged” ticks bit the runner in the days before they were discovered. 

### 2.2. Data Cleaning

We only included in the analysis the data associated with a correct bib number and racing round number. People who were already bitten during the competition might be more motivated to participate in the following days, or some people might participate only when bitten. Therefore, the participation in the study differed among the orienteers that were bitten by ticks, and this can bias the estimated proportion of orienteers bitten by ticks. We tested this hypothesis by modelling the participation of orienteers for a given racing round in the “banner study”—i.e., reporting their tick bite on the banner—using a logistic GLM (generalized linear model with a binomial distribution and a logit link, with the binary response variable: participation = 1; no participation = 0) according to the age class, gender, the number of times the person reported being bitten by ticks during the previous racing rounds, and the number of times they previously participated in the study. A model was run independently for each racing round and only included the orienteers that wrote at least once on the banner. The results showed that the probability of writing on the banner for a racing round was not influenced by age and gender and was lower for the persons bitten only one or two times during the previous racing rounds than those never bitten by ticks (Table S1). These results suggest that some orienteers only participated on the day they were bitten, which was confirmed by the high proportion of tick bites in orienteers that participated only once or twice in the study (64%).

As a consequence, we can consider that those who wrote on the banner four times or more were not influenced by the fact that they were bitten by a tick. Therefore, we used only one dataset for the subsequent analysis: orienteers who wrote at least four times on the banner (hereinafter referred to as the “restricted banner dataset”), as summarized in Figure 2. An analysis of the overall dataset, i.e., orienteers who participated at least once in the study by writing on the banner or by submitting ticks, can be found in Appendix A.

### 2.3. Participation in the Study

We calculated the participation rate in the study per racing round by dividing the number of orienteer-days that participated in any aspect of the study (by reporting information on the banner and/or by submitting ticks) by the number of orienteer-days running the race. An orienteer-day refers to one orienteer during one day, without considering if the orienteer participated in the study on another day (Figure 2). We then estimated the specific participation rate in the “banner study” per racing round as the proportion of orienteer-days running the race who reported information on the banner only, by considering only those who wrote at least four times on the banner. Similarly, we calculated the overall participation rate in the study and on the banner study for all the racings rounds by calculating the proportion of orienteers who participated at least in one racing round in the study or the banner study. To assess potential biases of recruitment, we compared age and gender profile between participating orienteers and the overall orienteer population of the competition using the following age classes: (7–15], (15–20], (20–35], (35–50], (50–65], >65. 

### 2.4. Frequency of Tick Bites among Orienteers

We estimated the proportion of orienteers bitten by ticks (=prevalence of tick bites) during a racing round by using the information relative to tick bites (tick bite/no tick bite) in the restricted banner dataset: we divided the number of orienteers who reported to have been bitten by the total number of orienteers who participated four times and more on the banner study. Exact confidence intervals of 95% (95% CI) were calculated using the binomial distribution. The prevalence of tick bites per racing round was compared with a Chi-squared test.

In order to characterize the space–time exposure of orienteers to tick bites, we calculated two exposure indices of tick-bites for each racing round that took into account the overall time spent and the distance travelled in the forest when using the restricted banner dataset. We first calculated the number of orienteers bitten by ticks among 100 orienteers walking one kilometer (number of orienteers bitten by ticks per 100 orienteer-kilometers): the number of orienteers bitten by ticks was divided by the cumulated kilometers walked by all orienteers participating on the banner study, using the minimum distance given by the competition organizers of each course per category, to produce a number of bites per orienteer per kilometer, which was then multiplied by 100. Secondly, we calculated the number of orienteers bitten by ticks among 100 orienteers walking for hour (number of orienteers bitten by ticks per 100 orienteer-hours): the number of orienteers bitten by ticks was divided by the cumulated time recorded for the completion of the racing round by all orienteers to produce the number of bites per orienteer per hour, which was then multiplied by 100. 

### 2.5. Analysis of Submitted Ticks: Frequency of Engorged Ticks and of Larvae, Nymphs, and Adults

The proportion of orienteers with engorged ticks was an indicator of the frequency of undetected tick bites after a forest activity, which may influence the risk of transmission of a tick-borne pathogen as this risk increased with the feeding duration of ticks. We were also interested to assess whether the proportion of orienteers with engorged ticks increased with each racing day and would influence the estimated prevalence of tick bites per racing round. Therefore, we calculated this proportion per racing round among the orienteers who submitted ticks and tested the racing round effect using a Chi-squared test. 

To study the infestation by larvae, nymph, and adult ticks of orienteers who submitted ticks per racing round, we excluded from the analysis the ticks that were found engorged. We tested the effect of racing round on the proportion of orienteer-days and on the mean infestation of orienteer-days for each stage using a Chi-squared test and a one-way ANOVA (ANalysis Of VAriance), respectively, with post-hoc comparisons performed using Tukey’s HSD test. 

### 2.6. Frequency of Repellent Use and Effect on the Prevalence of Tick Infestation

We estimated the proportion of orienteer-days per racing round and the overall proportion of orienteers for all racing rounds that used repellent at least once by using the restrictive banner dataset. Their associated 95% CI were calculated using the binomial distribution. The effect of age and gender on the use of repellent was tested in modelling the use of a repellent at least once with a logistic GLM as a function of age group, gender, and the interaction between age group and gender. We tested the effect of the use of a repellent on the prevalence of tick infestation of orienteer-days by using a Chi-squared test.

Statistical analysis and figure drawing were performed using R v. 3.5.0 [44].

## 3. Results

### 3.1. Participation in the Study

Fifty-one orienteer-days misspelled their race bib number or the race location in the form associated with the collected tick tube and were therefore excluded from the analysis. In total, 2693 orienteer-days including 710 unique individuals (48% of the total orienteers) participated in the study by reporting information on the banner and/or by submitting ticks. This included 676 orienteer-days that submitted ticks, of which 187 orienteer-days only submitted ticks without reporting on the banner (Figure 2). More than 686 orienteer-days reported being bitten by ticks on the banner, and 489 (71%) submitted ticks. Overall, 410 unique individuals participated in the study by writing on the banner at least four times, which represented a total of 2076 orienteer-days for the restricted banner dataset (Figure 2).

**Figure 3:**
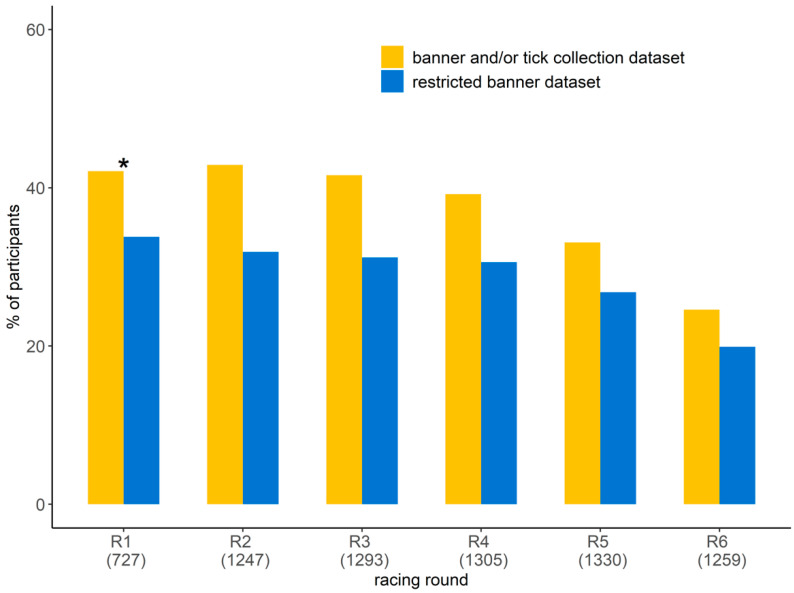
**Participation rates in the study per racing round**: proportion of orienteers participating in the study per racing round by reporting tick information on the banner and/or by submitting ticks (in yellow) and the proportion of orienteers who reported tick information on the banner considering only those that reported tick information on the banner at least four times (i.e., the restricted banner dataset, in blue). The number in parentheses represents the number of orienteers participating in the competition. * Racing round 1: no ticks were collected.

The number of orienteers participating in the study ranged from 310 to 538 per race, resulting in a total participation rate per racing round ranging from 24.6% and 46.1% (Figure 3). The participation rate was lower for the last two racing rounds (races 5 and 6). Half of the participants (56.8%) ran the six racing rounds (Table 1). The proportion of orienteers participating for the first time in the study was less than 10% from racing round 3 to 6. By considering only the orienteers that reported information on the banner at least four times, the participation rate varied from 19.9% to 33.8% per racing round, with a lower proportion in the last two racing rounds (5 and 6). The participants were 55.1% male and 44.9% female, and this was not statistically different from the proportion of male and female among orienteers (χ^2^ = 2.6, df = 1, *p* = 0.1, Table 1). Their age ranged from 8 to 82 years with the 20–35 and >65 age groups being scarcer (Table 1). The distribution per age group was not statistically different between the participants in the study and the orienteers participating in the competition (χ^2^ = 4.1, df = 5, *p* = 0.5). There was no statistical difference in age group and gender distribution between the participants in the study and those participating in the competition for each racing round (Table S2).

### 3.2. Frequency of Tick Bites among Orienteers

**Figure 4:**
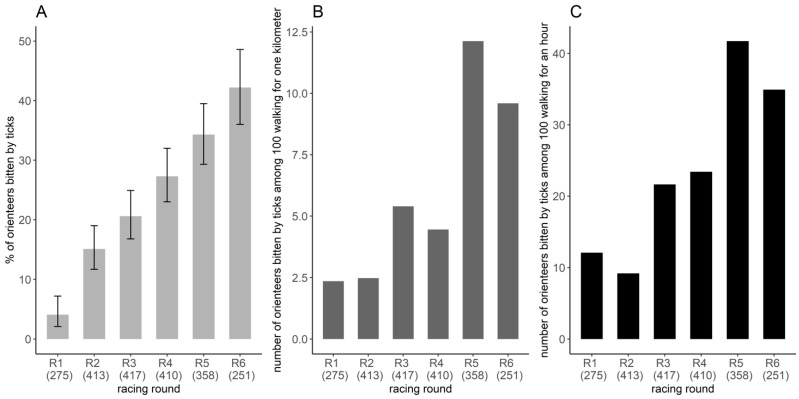
Frequency of tick bites per racing round. (**A**) Proportion of orienteers bitten by ticks per racing round and its 95% confidence interval, estimated by the number of orienteers reporting tick-bites on the banner divided by the number of orienteers reporting on the banner, considering only the orienteers that participated four times or more (restricted banner dataset). Space–time exposure index of the number of orienteers bitten by ticks among 100 orienteers walking (**B**) one kilometer or for one hour (**C**). The number in parentheses represents the number of orienteers participating in the study.

Based on the restricted banner dataset (*n* = 2076 orienteer-days), 23.6% of orienteer-days were bitten by ticks. The estimated proportion of orienteer-days bitten by ticks varied significantly between racing rounds (Figure 4A). This proportion was the lowest for the first racing round, with 4.1% (CI_95%_: 2.1–7.2%) of orienteer-days bitten by ticks and then varied from 15.1% (CI_95%_: 11.7–19.0%) to 42.2% (CI_95%_: 36.0–48.6%) (χ^2^ = 148.5, df = 5, *p* < 0.001), with the highest proportion in racing rounds 5 and 6 (Figure 4A). Overall, among the 410 participants who wrote on the banner at least four times, 62.4% (256/410) reported to have been bitten by ticks during at least one racing round over the six days.

During the competition, the exposure indices varied from 2.4 to 12.1 orienteers bitten by ticks per 100 orienteers walking 1 km and from 9.2 to 41.7 orienteers bitten by ticks per 100 orienteers walking for 1 h (Figure 4B,C). Exposure was higher in the last two racing rounds, 5 and 6, and lower in racing rounds 1 and 2. The order of the values per racing round differed slightly between the two exposure indices (Figure 4B,C).

### 3.3. Analysis of Submitted Ticks: Frequency of Engorged Ticks and of Larvae, Nymphs, and Adults

Overall, 1651 ticks were submitted by 676 orienteer-days during racing rounds 2 to 6, representing 448 unique orienteers. All but one *Dermacentor* spp. female were *I. ricinus*. There were 564 larvae (34.2%), 1062 nymphs (64.3%), and 24 females (1.4%) (Table 2). Of these, two (0.4%) larvae, 80 (7.6%) nymphs, and one female (4.1%) were engorged (Table 2), meaning that they did not bite during the day of collection. The proportion of orienteer-days with engorged larvae (1.3%, *n* = 158) was significantly lower than those with engorged nymphs (11.9%, *n* = 607; Fisher’s test, *p* < 0.001). Among the 676 orienteer-days having submitted ticks, 23.4%, 89.9%, and 3.3% were bitten by respective larvae, nymphs, and females, and 8.7% were bitten only by larvae. The engorged ticks were submitted by 75 orienteer-days (11.1%), which represented 75 unique orienteers (16.7%). The proportion of orienteers submitting engorged ticks was not significantly different between racing rounds (χ^2^ = 2.1, df = 4, *p* = 0.7, Table 2).

Regarding submitted non-engorged ticks (i.e., ticks that bit during the same racing day they were collected), significantly more orienteers were bitten by larvae on racing round 6 compared to the previous ones (χ^2^ = 17.1, df = 4, *p* = 0.001), while there was no significant difference in the proportion of orienteers bitten by nymphs (χ^2^ = 3.7, df = 4, *p* = 0.44) and females (Fisher’s exact test, *p* = 0.16) between racing rounds (Table 3). In parallel, the mean number of larvae and nymphs per orienteer bitten by ticks was also significantly higher in racing rounds 5 and 6 for larvae (one-way ANOVA, F(4616) = 4.57, *p* = 0.001 and post-hoc comparisons using Tukey HSD test) and for nymphs (one-way ANOVA, F(4616) = 7.94, *p* < 0.001 and post-hoc comparisons using Tukey HSD test). Overall, 33.2% and 14.7% of orienteer-days were bitten by more than two and three nymphs, respectively; 11.8% and 8.0% were bitten by more than two and three larvae, respectively (Figure S1). Larvae were highly aggregated on the same persons, with 85% of larvae found attached on 11.8% of orienteers-days and a mean infestation of 3.6 larvae per person bitten per larvae. Nymphs were less aggregated, with 58.4% of nymphs found on 33.2% of orienteer-days and a mean infestation of 1.8 nymphs per person bitten per larvae. The maximum number of non-engorged larvae, nymphs, and females found on one individual was 45, 14, and 2, respectively (Figure S1). The highest numbers of ticks found biting the same orienteer were 45 larvae and 10 nymphs on the 5th day and 17 larvae and 6 nymphs the day after. There was a positive correlation between the number of larvae and nymphs infesting orienteers (Pearson’s correlation test: r = 0.39, *p* < 0.001).

### 3.4. Frequency of Repellent Use

**Figure 5:**
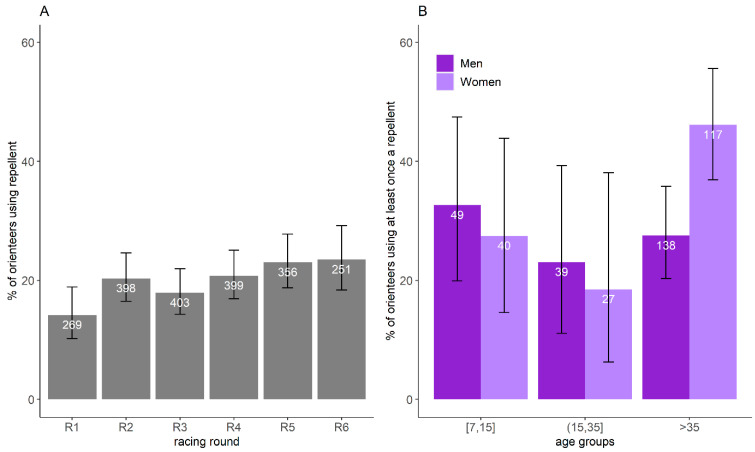
**Proportion of orienteers using repellent and its confidence interval of 95%** per racing round (**A**) and per age group and gender (**B**), estimated by the restricted banner dataset. The number represents the number of orienteers reporting information on repellent use in the restricted banner dataset.

Based on the restrictive banner dataset, 20.0% of orienteer-days participating in the study (CI_95%_:18.3–21.7%, *n* = 2074) used a repellent. The proportion did not vary significantly with the racing round (χ^2^ = 10.9, df = 5, *p* = 0.06) (Figure 5A). Overall, 32.4% of orienteers (CI_95%_: 27.9–37.2%) used a repellent for at least one racing round, and 8.5% systematically used them. GLM results showed that only gender (*p* = 0.05) influenced the probability to have used a repellent at least once over the six days. When orienteers older and younger than 35 years old were grouped within the same age group in the model, GLM results showed that the age groups (*p* = 0.02), gender (*p* = 0.008), and their interactions (*p* = 0.03) were significant. The use of repellent was less frequent for women younger than 35 years old than those older than 35 years old (≤35 years old: 23.9%, CI_95%_: 14.3–35.9%; >35 years old: 46.2%, CI_95%_: 36.9–55.6%) and was more frequent for women older than 35 years old compared to men of the same age group (women: 46.2%, CI_95%_: 36.9–55.6%; men: 27.5%, CI_95%_: 20.3–35.8%) (Figure 5B).

In total, 19.7% (CI_95%_: 16.0–24.0%) of the orienteers using repellent were bitten by ticks, which was slightly but significantly lower than the proportion of orienteers not using a repellent that were bitten by ticks (24.6%, CI_95%_: 22.6–26.8%, χ^2^ = 4.1, df = 1, *p* = 0.04).

## 4. Discussion

Mass-participation outdoor events are good opportunities to study risks associated with ticks [29,42]. The overall increasing concern and awareness toward tick-borne disease risk in the general population and more specifically in high risk populations [20,36,39,45,46] are expected to make people more prone to participate in a study related to tick and tick-borne disease risks. The high participation of volunteers in our study confirms this hypothesis: half of the orienteers participated at least once during the six days of the competition, representing 37% of the total orienteer-days. This shows that citizen science approaches are particularly suited to work with this type of population.

Our results confirmed that orienteering competition is an especially high-risk activity for tick bites [35,36,37,39], since a very high proportion of orienteers were bitten by ticks during the competition. Our estimates are that 62% of the orienteers were bitten by ticks over the six days of the competition, varying daily from 16% (CI_95%_: 12.6–19.9%) to 42.2% (CI_95%_: 36.0–48.6%). We cannot exclude that people who are generally more prone to being bitten by ticks were more motivated to participate in this study for all racing rounds. If this were the case, the proportion of orienteers bitten by ticks might be slightly over-estimated in our study. The real proportion of orienteers bitten by ticks is probably between the proportion of orienteers who reported to have been bitten among all the competitors—which is a “minimal proportion”, largely below the real proportion of orienteers bitten by ticks (see the results in Appendix A)—and the proportion of orienteers who reported to have been bitten among those who participated at least four times in the study. The expected proportion of orienteers bitten by ticks is therefore between 33% and 62% during the six days of the competition and between 10% to 42% per day, which is in either case very high. For comparison with general populations of other countries, a range of 45–60% of respondents reported finding attached ticks over a preceding 12-month period in surveys conducted in Scandinavia [47] and in North-West Italy [48]. In endemic areas of Lyme disease in USA, this proportion was in the range of 10–37% [25], and in France it has been estimated that only 4.1% of the general population was bitten over a period of 12 months [46]. In another mass-participation event in Scotland, a two-day mountain marathon in the highlands included 626 competitors [29]; they observed that the minimal proportion of competitors bitten by ticks each day was 8–14%. This is similar to the minimal proportion of competitors bitten by ticks per day in our study (10–15%). However, this measure depends on the participation rate, which was probably lower in our study compared to the study in Scotland given that the number of participants in the competition was the double that in the marathon in Scotland (1491 vs. 626 competitors). Hence, the proportion of competitors bitten by ticks was probably higher in our study.

When considering the space–time exposure to tick bites during the competition, the exposure indices were between 2.4 and 12.1 orienteers bitten by ticks per 100 orienteers walking 1 km and between 9.2 and 41.7 orienteers bitten by ticks per 100 orienteers walking for 1 h. Given that 90% of orienteer-days were bitten by nymphs, these values are close to the space–time exposure indices of orienteers bitten by nymphs. Higher values were found in the two last racing rounds. This is consistent with the higher number of ticks, larvae, and nymphs per person bitten by ticks observed in the two last racing rounds. The difference observed is probably due to local variations of the density of questing ticks in the environment that can be important even at closed locations according to vegetation, soil or the density of hosts [49,50,51,52]. The exposures indices we found were higher than those observed by Hall et al. [29] during the 2-day marathon in Scotland. They estimated that people were exposed to tick-bites for ≈32 h per competitor including the camping evening. This resulted in 0.7 competitors bitten by ticks (0.49 by nymphs) per 100 competitors exposed for 1 h. If we considered only the exposure to tick-infested areas during the 20–40 km competition of 8–10 h (i.e., without the camping evening), 0.5–1.0 competitors per 100 competitors walking 1 km and 2.1–2.6 per 100 competitors walking for 1 h were bitten by ticks. These values are much lower than those estimated in our study. This can be partly explained by a lower questing tick density in the environment where the marathon in Scotland took place compared to our study site. Both competitions occurred in June—early July during the peak activity of immature ticks, larvae, and nymphs. We did not measure the density of questing ticks during the race, unlike Hall et al., who estimated around 98 nymphs/100 m^2^ in three sites. However, multi-annual monitoring of questing tick density in forest areas of the same region [53,54] (CLIMATICK and CCEID project unpublished data) showed that 2018 was among the years with the highest density of questing nymphs. Indeed, the density of questing nymphs reached 140–190 nymphs/100 m^2^ in these forests in June–July 2018. Local variations in the questing density of ticks are probably not the sole factors explaining the difference in the exposure to tick bites measured between the two surveys. The difference can also be explained by the type of activity [26]: the participants of the marathon were running along trails, whereas orienteers have to search for orientation beacons by walking through vegetation; this is riskier behavior to be in contact with ticks and to get bitten. Thus, the values of the space–time exposure to tick bites estimated in our study probably represent higher values for forest activities considering (i) orienteering behavior is particularly at risk for tick bites, (ii) the race occurred during the period of the peak activity of immature ticks, and (iii) 2018 was a year with high questing nymph density.

Most of the ticks found in our study were nymphs of *I. ricinus* and had an aggregated distribution on the same individuals. Nymphs of *I. ricinus* are generally considered to be the most important life stage for pathogen transmission due to their abundance and their small and not-easy-to detect size [25,55]. In our study, nymphs were mostly found alone on the body, but in 33% of cases, from two to fourteen nymphs were found together. Larvae, representing 34% of collected ticks, were mostly found attached on the body simultaneously with nymphs and were found alone in only 9% of orienteer-days. Detecting a nymph might increase the thoroughness of the tick check and thus increase the chance of detecting a larva if one is attached. We found very few adults compared to other observations of ticks biting humans [9,10,12,13]. This is not surprising since the proportion of each tick stage in the environment varies according to the period of the year and the location. We observed that ticks displayed an aggregated distribution on orienteers, especially for larvae. This is similar to the tick distribution on their natural hosts, where around 80% of the ticks feed on 20% of the hosts [56,57,58,59]. This is consistent with the observations during the mountain marathon in the Scottish Highlands [29]. This might be explained by the clustered distribution of host-seeking ticks in the environment, which is known to be more important for larvae than nymphs [52,55,60]. Individual factors might also influence the attachment of ticks on people, although there is no scientific evidence. Indeed, ticks are commonly reported to always bite the same people within a family. On the one hand, multiple tick bites on the body might increase the individual risk of transmission of pathogens if the ticks are not detected early enough. On the other hand, repeated tick bites can result in hypersensitivity and increase itching in humans, leading to increased detection of attached ticks and hence reduced risk of acquisition of tick-borne diseases [25,61].

The risk of not detecting a tick bite was relatively high in our study, considering that the communication we made at the stand to remove attached ticks might have stimulated more people to check for ticks after their runs. Despite this stimulation, 16.7% of orienteers bitten by ticks had engorged ticks, meaning that they did not detect and remove their ticks after their racing round. This suggests that around 10% of orienteers participating in the competition might have failed to detect a tick just after the racing round, when considering that 62.4% of orienteers were bitten at least once over the six days of the competition. Detection of attached ticks can be biased by the size of the ticks, with *Ixodes ricinus* larvae measuring less than 1 mm and nymphs less than 1.5 mm. In our study, most engorged ticks were nymphs, followed by larvae. Only one person removed an engorged female. To our knowledge, this represents the first attempt to quantify the risk of not detecting a tick bite immediately after exposure to tick-infested areas. We do not know how well orienteers that found engorged ticks performed their tick check or if they did it at all, but these results show that one tick check after a dangerous activity is probably not enough, and that a tick check should be performed at least one other time as soon as possible on the same day of the risky activity. In the future, we would recommend the installation of a stand to raise awareness of the risk related to ticks and to remove ticks in mass-participation outdoor events, with awareness messages for multiple tick checks.

While repellents are a highly effective personal protective measure against ticks [24,62,63,64,65], in our study, surprisingly, a high proportion of orienteer-days that used a repellent was bitten by ticks (19.7%). This was only 5% less than orienteer-days that did not use one. As we did not ask for the name of the repellent or for the way it was used, we do not know what people used as a repellent or how they used it, except for one woman who was bitten and wrote that she used moisturizing cream as a repellent. It is thus possible that people who used repellents and were still bitten by ticks did not use an efficient repellent or that they did not use it correctly. It is also possible that the repellent used was not 100% effective or did not last enough for the duration of the run [62,66,67,68]. Our results indicate that around 20% of orienteers did not protect themselves as efficiently as they thought they were by using a repellent. The proportion of orienteers using repellent during at least one race was low (34.2%), and only a few used a repellent in each race (8.5%). This could be due to bias with people not declaring their use of a repellent every time. The overall proportion was still low, especially for such a high-risk population. It is not different than the use of repellent reported by people who felt fairly exposed to ticks in France [46]. This is not what we should expect from people who are heavily bitten by ticks (33 to 64% of orienteers) since high-risk populations are usually more aware of tick-associated risks and more prone to use personal protective measures against tick bites [22,45,69,70]. It would be interesting to study why orienteers in France are not more prone to use repellents. This could be due to a reluctance to use chemical products, as it was mentioned by several people participating in the CiTIQUE program, or by a lack of proper messages on which repellent to use and how to use it. We found that the proportion of orienteers using repellent at least once was higher for women older than 35 years old (46.2%). This confirms the results of several studies that have shown that there are differences in the acceptance of protective measures against tick risk in the population, with women usually more inclined to protect themselves than other categories [22,45,71,72]. 

## 5. Conclusions

Our study confirms that orienteers are a high-risk population for tick bites, with 62.4% of the orienteers bitten by ticks over the six days of the competition. Using a citizen science approach, this event allowed us to provide quantitative data on the frequency of tick bites on orienteers according to walking distance and time spent in the forest, on the use of repellent, and for the first time, on the proportion of tick bites that were not detected immediately after exposure to tick-infested areas. Space–time exposure of orienteers to tick bites was high compared to other studies (general population or marathon), but it can be explained by the high density of ticks observed this year and by the risky behaviors that are involved in orienteering activities, resulting in high contacts with ticks. Despite advertising on tick risk before and during the competition, the use of personal protective measures against ticks—self tick checks, the way of using a repellent or what people think to be a repellent—did not appear to be completely efficient. These results show that prevention should be increased for orienteers and other high-risk populations, with a focus on the need to performed tick checks several times after a risky event, and the need to provide more information on tick repellents and how to use them. This study’s collected data are rare and may be useful to provide parameters for modelling human exposure to ticks and tick-borne pathogens.

## Figures and Tables

**Table 1:** Characteristics of the whole population of orienteers and of the population of study (i.e., the orienteers who reported tick information on the banner and/or submitted ticks) related to gender, age, and racing round number.

	Orienteers (*n* = 1491)		Participation by Writing on the Banner or by Submitting Ticks (*n* = 710)		Participation by Writing on the Banner Only ≥ 4 Times (*n* = 410)	
	*n*	%	*n*	%	*n*	%
**Gender**						
Men	772	59.6	395	55.6	226	55.1
Women	524	40.3	315	44.4	184	44.9
*Unknown*	*195*					
**Age**						
(7,15]	268	20.4	166	23.4	89	21.7
(15,20]	137	10.5	73	10.3	32	7.8
(20,35]	86	6.6	53	7.5	34	8.3
(35,50]	340	26.0	186	26.2	105	25.6
(50,65]	336	25.7	173	24.4	110	26.8
(65,82]	141	10.8	59	8.3	40	9.8
*Unknown*	*183*		*0*		*0*	
**No. racing rounds run**						
1	81	5.4	3	0.4	0	0
2	55	3.7	14	2.0	0	0
3	87	5.8	20	2.8	0	0
4	64	4.3	19	2.7	6	1.5
5	456	30.6	251	35.4	135	32.9
6	748	50.2	403	56.8	269	65.6
**No. racing rounds with a participation in the study**						
1	-	-	122	17.2	0	0
2	-	-	87	12.3	0	0
3	-	-	72	10.1	0	0
4	-	-	125	17.6	122	29.8
5	-	-	143	20.1	140	34.1
6	-	-	161	22.7	148	36.1

**Table 2:** Data summary of the total number of ticks and substantially engorged ticks submitted by orienteers participating in the study. Tot.: Total, Eng.: engorged.

Racing Round	No. of Orienteers with Ticks		No. of Ticks Collected					
	Tot.	Eng.	Larvae		Nymphs		Females	
			Tot.	Eng. (%)	Tot.	Eng. (%)	Tot.	Eng. (%)
**2**	**85**	8 (9.5%)	34	0	93	9 (9.7%)	5	0
3	112	13 (11.6%)	55	0	125	12 (9.6%)	3	1 (33.3%)
4	154	14 (9.1%)	39	0	229	15 (6.6%)	7	0
5	176	24 (13.6%)	228	1 (0.4%)	329	28 (8.5%)	8	0
6	149	16 (10.7%)	208	1 (0.5%)	286	16 (5.6%)	1	0
Total runners-days	676	75 (11.1%)	564	2 (0.4%)	1062	80 (7.5%)	24	1 (4.2%)

**Table 3:** Data summary of the infestation of orienteers by non-engorged ticks per racing round. Number and proportion of orienteers submitting non-engorged larvae, nymphs, and females per racing round and mean number of ticks per orienteer-days.

Racing Round	No. of Orienteers with Non-Engorged Ticks	No (%) of Orienteers with Non-Engorged			Mean Number of Non-Engorged (L,N,F) per Orienteer Bitten by Ticks		
		Larvae	Nymphs	Female	Larvae	Nymphs	Female
**2**	79	13 (16.4%)	67 (84.8%)	3 (3.8%)	0.43	1.06	0.06
3	102	24 (23.5%)	86 (84.3%)	2 (2.0%)	0.54	1.11	0.02
4	144	24 (16.7%)	132 (91.7%)	7 (4.9 %)	0.27	1.49	0.05
5	161	47 (29.2%)	144 (89.4%)	8 (5.0%)	1.41	1.87	0.05
6	138	48 (34.8%)	125 (90.6%)	1 (0.7%)	1.50	1.96	0.01
**Total orienteer-days**	624	156 (25.0%)	554 (88.8%)	21 (3.4%)	0.90	1.57	0.04

**Figure A1:**
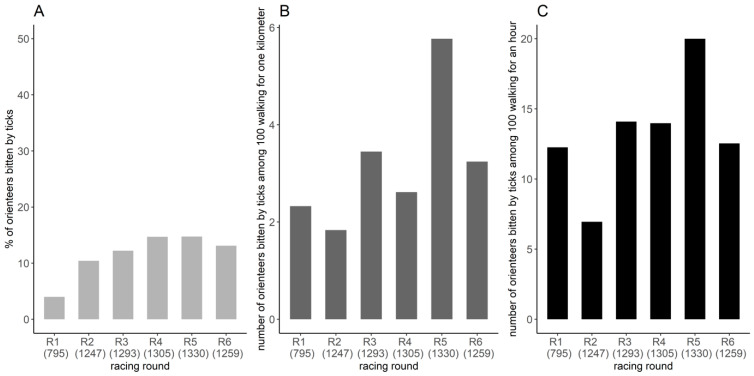
Frequency of tick bites per racing round. (**A**) Minimal proportion of orienteers bitten by ticks per racing round and its 95% confidence interval, estimated by the number of orienteers reporting tick-bites on the banner and by submitting ticks divided by the number of orienteers running the competition. Exposure index of the minimal number of orienteers bitten by ticks among 100 orienteers walking (**B**) one kilometer or for one hour (**C**). The number in parentheses represents the number of orienteers running the racing round.

**Figure A2:**
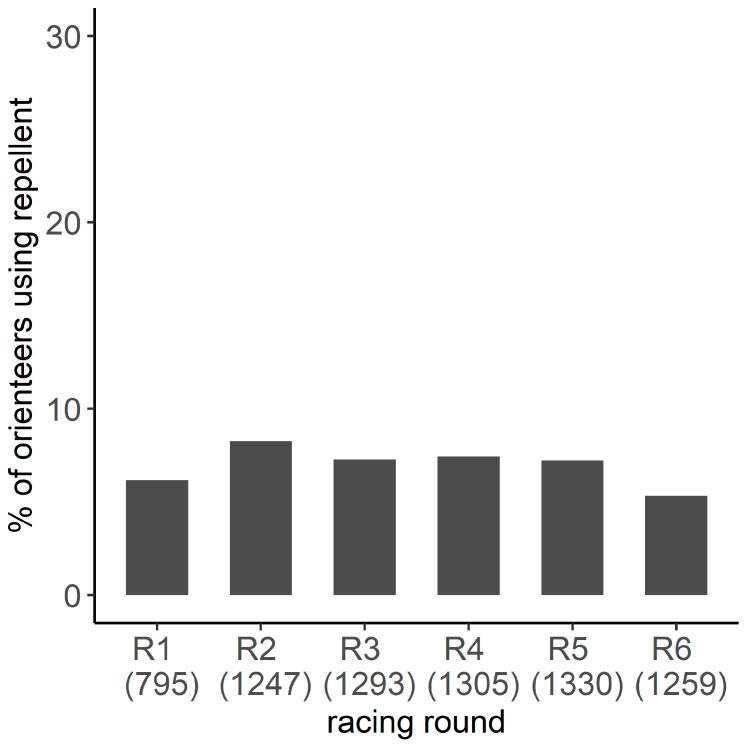
Minimum proportion of orienteers using repellent per racing round. The number represents the number of orienteers participating in the competition.

## Data Availability

Data are available on request from the authors.

## Supplementary Materials

The following are available online at https://www.mdpi.com/1660-4601/18/6/3161/s1, Figure S1: Histogram of the number of larvae, nymphs, and females per orienteer-day having submitted ticks (*n* = 626). Table S1: Odds ratio (OR) and their associated 95% confidence intervals (CI) obtained from each logistic GLM of the probability of reporting tick information on the banner for a given racing round for orienteers that previously participated in the study. A model was run independently for each racing round. Table S2: Characteristics of the population of orienteers participating in the competition for each racing round and those who reported tick information on the banner at least four times.

## Appendix A

We present here the results obtained for the data of orienteers that participated at least once in the study by writing on the banner or by submitting ticks.

### Appendix A.1. Materiel & Methods

#### Appendix A.1.1. Frequency of Tick Bites among Orienteers

The overall proportion of orienteers who reported to have been bitten by ticks during a racing round was calculated as follows: the overall number of persons who reported to have been bitten by ticks either on the banner or by submitting ticks was divided by the overall number of participants in the racing round. This proportion gave an idea of the minimal proportion of orienteers that were bitten by ticks during the racing round.

The minimal number of orienteers bitten by ticks among 100 orienteers walking one kilometer was calculated with the number of orienteers reporting having bitten by ticks on the banner or by submitting ticks multiplied by 1000 divided by the cumulated kilometers walked by all orienteers participating in the competition, using the minimum distance of each course per category. Similarly, the minimal number of orienteers bitten by ticks among 100 orienteers walking during one hour was calculated with the number of orienteers bitten by ticks multiplied by 100 divided by the accumulated time recorded for the completion of the racing round by all orienteers.

#### Appendix A.1.2. Frequency of Repellent Use

We calculated the minimum proportion of orienteers that used a repellent during a racing round: we divided the overall number of persons who reported to have used a repellent either on the banner or on the form when submitting ticks by the overall number of participants in the racing round. Similarly, we calculated the minimum proportion of orienteers who used a repellent at least once among all racing rounds and those who systematically used a repellent for all racing rounds.

### Appendix A.2. Results

#### Appendix A.2.1. Frequency of Tick Bites among Orienteers

The minimal proportion of orienteer-days bitten by ticks calculated by using all tick-bite reports among all orienteer-days was quasi constant for the racing rounds from 2 to 6, varying from 10.4% to 14.7% (Figure A1). The first racing round displayed a low minimal proportion of 4.0% but cannot be compared to the others because no ticks were collected.

During the competition, the minimal exposure index of tick-bites varied from 1.8 to 5.8 orienteers bitten by ticks per 100 orienteers walking 1 km and from 6.9 to 20.0 orienteers bitten by ticks per 100 orienteers walking for 1 h (Figure A1).

#### Appendix A.2.2. Frequency of Repellent Use

Overall, the minimum proportion of orienteer-days using a repellent was 18.8%. The proportion varied from 5.3% to 8.2% according to the racing round (Figure A2). Overall, a minimum of 27.3% of orienteers used a repellent during at least one racing round, and a minimum of 10.1% systematically used them.
