# Supplementary material for: Are Orienteers Protected Enough against Tick Bites? Estimating Human Exposure to Tick Bites through a Participative Science Survey during an Orienteering Competition
Source: Int J Environ Res Public Health. 2021 Mar 18;18(6):3161. doi: 10.3390/ijerph18063161 (PMC8003242; doi:10.3390/ijerph18063161)
Supplement: Supplementary file 1 [file ijerph-18-03161-s001.pdf]

International Journal of
*Environmental Research and Public Health*

MDPI

# Are Orienteers Protected Enough Against Tick Bites? Estimating Human Exposure to Tick Bites Through a Participative Science Survey During an Orienteering Competition- SUPPLEMENTARY MATERIALS

**Appendix A**

We presented here the results obtained with the data of orienteers who participated at least once in the study by writing on the banner or by submitting ticks.

**Materiel & Methods**

*Frequency of tick bites among orienteers*

The overall proportion of orienteers who reported to have been bitten by ticks during a racing round was calculated as follows: the overall number of persons who reported to have been bitten by ticks either on the banner or by submitting ticks was divided by the overall number of participants to the racing round. This proportion gives an idea of the minimal proportion of orienteers who have been bitten by ticks during the racing round.

The minimal number of orienteers bitten by ticks among 100 orienteers walking during one kilometer, was calculated with the number of orienteers reporting having bitten by ticks on the banner or by submitting ticks multiplied by 1000 divided by the cumulated kilometers walked by all orienteers participating in the competition, using the minimum distance of each course per category. Similarly, the minimal number of orienteers bitten by ticks among 100 orienteers walking during one hour was calculated with the number of orienteers bitten by ticks multiplied by 100 divided by the cumulated time recorded for the completion of the racing round by all orienteers.

*Frequency of repellent use*

We calculated the minimum proportion of orienteers who used a repellent during a racing round: we divided the overall number of persons who reported to have used a repellent either on the banner or on the form when submitting ticks by the overall number of participants to the racing round. Similarly, we calculated the minimum proportion of orienteers who used at least once a repellent among all racing rounds and those who used systematically a repellent for all racing rounds.

**Results**

*Frequency of tick bites among orienteers*

The minimal proportion of orienteer-days bitten by ticks calculated by using all tick-bite reports among all orienteer-days was quasi constant for the racing round from 2 to 6 varying from 10.4% to 14.7% (Figure A1). The first racing round displayed a low minimal proportion of 4.0% but cannot be compared to the others because no ticks were collected.

During the competition, the minimal exposure index of tick-bites varied from 1.8 to 5.8 orienteers bitten by ticks per 100 orienteers walking 1 km and from 6.9 to 20.0 orienteers bitten by ticks per 100 orienteers walking 1 hour (Figure A1).

*Frequency of repellent use*

Overall, the minimum proportion of orienteer-days using a repellent was 18.8%. The proportion varied from 5.3% to 8.2% according to the racing round (Figure A2). Overall, a minimum of 27.3% of orienteers used a repellent for at least one racing round and a minimum of 10.1% systematically used them.

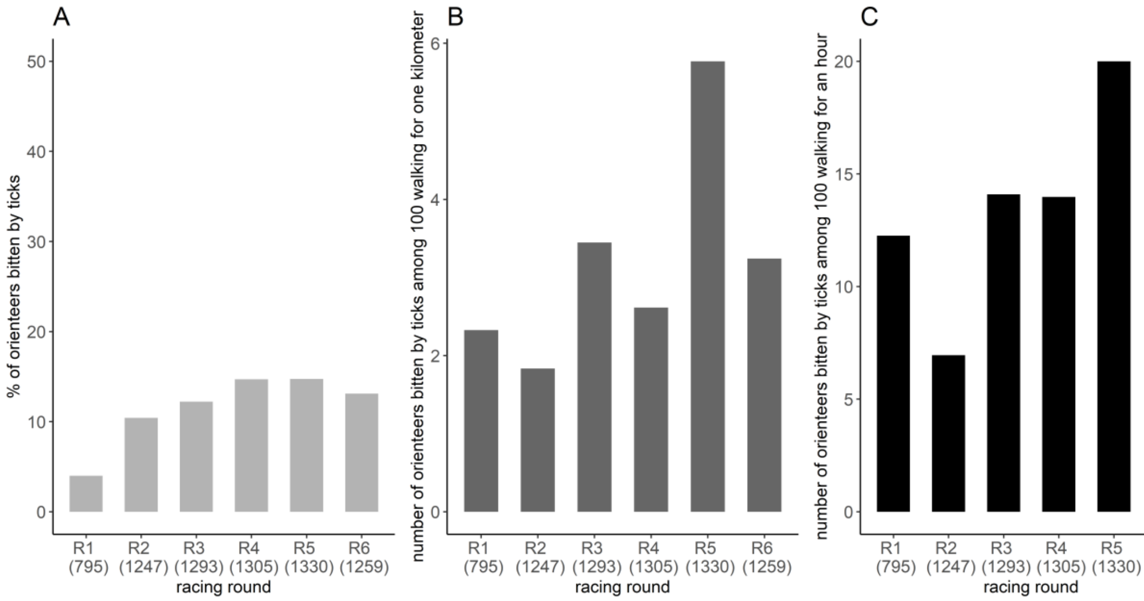


**Figure A1.** Frequency of tick bites per racing round. (A) Minimal proportion of orienteers bitten by ticks per racing round and its 95% confidence interval, estimated by the number of orienteers reporting tick-bites on the banner and by submitting ticks divided by the number of orienteers running the competition; Exposure index of the minimal number of orienteers bitten by ticks among 100 orienteers walking for (B) one kilometer or for one hour (C). The number in brackets represent the number of orienteers running the racing round.

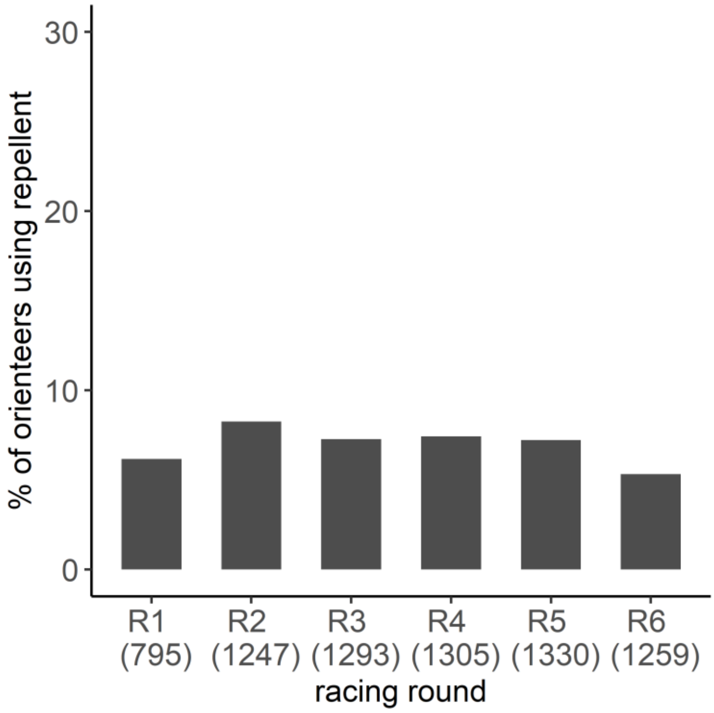


**Figure A2.** Minimum proportion of orienteers using repellent per racing round. The number represent the number of orienteers participating in the competition.

**Table S1.** Odds ratio (OR) and their associated 95% confidence intervals (CI) obtained from each logistic GLM of the probability of reporting tick information in the banner at a given racing round for orienteers having previously participated to the study. A model was run independently for each racing round.

| Variables | Racing round 2 (n=326) | Racing round 3 (n=519) | Racing round 4 (n=565) | Racing round 5 (n=592) | Racing round 6 (n=584) |
|---|---|---|---|---|---|
| **Gender** | | | | | |
| Ref = Women | | | | | |
| Men | 0,7 [0,2 - 1,9] | 0,7 [0,4 - 1,3] | 0,9 [0,4 - 2,1] | 0,9 [0,6 - 1,4] | 1,1 [0,7 - 1,6] |
| **Age groups** | | | | | |
| Ref = (35-50] | | | | | |
| [7-15] | 0,8 [0,2 - 1,9] | 0,6 [0,3 - 1,4] | 0,8 [0,4 - 1,6] | 1,0 [0,6 - 1,8] | 1,8 [1,0 - 3,1] |
| (15-20] | 0,7 [0,2 - 3,1] | 1,0 [0,3 - 3,5] | 0,7 [0,3 - 1,5] | 0,7 [0,3 - 1,4] | 0,7 [0,3 - 1,5] |
| (20-35] | 0,7 [0,1 - 4,0] | 1,7 [0,5 - 8,0] | 1,1 [0,4 - 3,4] | 1,4 [0,6 - 3,6] | 0,9 [0,4 - 2,0] |
| (50-65] | 2,5 [0,5 - 19,9] | 1,2 [0,5 - 3,0] | 0,9 [0,5 - 1,7] | 0,9 [0,5 - 1,6] | 1,6 [0,9 - 2,6] |
| >65 | 0,9 [0,1 - 19,2] | 1,3 [0,4 - 6,0] | 2,3 [0,8 - 7,6] | 0,8 [0,4 - 1,7] | 0,7 [0,3 - 1,4] |
| **Number of times a person was bitten before the race** | | | | | |
| Ref = 0 time | | | | | |
| 1 time | 0,2 [0,1 - 0,7]* | 0,2 [0,1-0,4]* | 0,8 [0,4 - 1,3] | 0,5 [0,3-0,8]* | 0,6 [0,4 - 0,9]* |
| 2 times | - | 0,1 [0,03 - 0,9]* | 0,7 [0,3 - 1,9] | 0,8 [0,4 - 1,5] | 0,9 [0,6 - 1,6] |
| ≥3 times | - | - | 0,5 [0,1 - 10,0] | 0,6 [0,2 - 1,8] | 1,8 [0,9 - 4,0] |
| **Number of times a person participated in the study before the race** | | | | | |
| Ref = 1 time | | | | | |
| 2 time | - | 3,2 [1,6 - 6,6]* | 5,8 [3,1 - 10,9]* | 2,0 [1,0 - 4,1] | 0,4 [0,1 - 1,0] |
| 3 times | - | - | 12,8 [6,8 - 25,1]* | 7,1 [3,8 - 13,5]* | 1,2 [0,5 - 2,6] |
| 4 times | - | - | - | 11,1 [6,1 - 20,8]* | 2,8 [1,4 - 5,5]* |
| 5 times | - | - | - | - | 8,2 [4,3 - 16,4]* |

**Table S2.** Characteristics of the population of orienteers for each racing round participating in the competition and those having reported tick information on the banner at least 4 times.

| | Racing round 1 | Racing round 2 | Racing round 3 | Racing round 4 | Racing round 5 | Racing round 6 |
|---|---|---|---|---|---|---|
| **Participants in the competition** | **n=795** | **n=1247** | **n=1293** | **n=1305** | **n=1330** | **n=1259** |
| **Gender** | | | | | | |
| Men | 470 (60.0) | 705 (59.9) | 712 (59.0) | 723 (59.8) | 724 (59,1) | 695 (59.9) |
| Women | 314 (40,0) | 471 (40.1) | 494 (41.0) | 487 (40.2) | 502 (40,9) | 465 (40.1) |
| *Unknown* | 5 | *71* | *87* | *95* | 104 | 99 |
| **Age** | **n (%)** | **n (%)** | **n (%)** | **n (%)** | **n (%)** | **n (%)** |
| [7,15] | 178(22,7) | 249 (20,9) | 251 (20,4) | 259 (21,1) | 259 (20,7) | 250 (21,3) |
| (15,20] | 91 (11,6) | 124 (10,4) | 129 (10,5) | 129 (10,5) | 141 (11,2) | 124 (10,5) |
| (20,35] | 55 (7,0) | 76 (6,4) | 78 (6,4) | 76 (6,2) | 82 (6,5) | 67 (5,7) |
| (35,50] | 183 (23,3) | 305 (25,6) | 308 (25,1) | 313 (25,5) | 325 (25,9) | 313 (26,6) |
| (50,65] | 205 (26,1) | 305 (25,6) | 321 (26,1) | 314 (25,6) | 312 (24,9) | 293 (24,9) |
| (65,100] | 72 (9,2) | 133 (11,1) | 134 (10,9) | 137 (11,1) | 135 (10,8) | 129 (11,0) |
| *Unknown* | *5* | *55* | *72* | *77* | *77* | *83* |
| **Participants writing on the banner ≥ 4 times** | **n=269** | **n=398** | **n=403** | **n=399** | **n=356** | **n=251** |
| **Gender** | | | | | | |
| Men | 150 (55.7) | 221 (55.5) | 223 (55.3) | 219 (54.9) | 197 (55.3) | 141 (56.2) |
| Women | 119 (44.3) | 177 (44.5) | 180 (44.7) | 180 (45.1) | 159 (44.7) | 110 (43.8) |
| **Age** | **n (%)** | **n (%)** | **n (%)** | **n (%)** | **n (%)** | **n (%)** |
| [7,15] | 61 (22,7) | 84 (21,1) | 85 (21,1) | 86 (21,6) | 79 (22,2) | 62 (24,7) |
| (15,20] | 25 (9,3) | 31 (7,8) | 32 (7,9) | 30 (7,5) | 24 (6,7) | 17 (6,8) |
| (20,35] | 25 (9,3) | 34 (8,5) | 34 (8,4) | 33 (8,3) | 29 (8,1) | 17 (6,8) |
| (35,50] | 67 (24,9) | 102 (25,6) | 103 (25,6) | 104 (26,1) | 94 (26,4) | 60 (23,9) |
| (50,65] | 71 (26,4) | 107 (26,9) | 110 (27,3) | 106 (26,6) | 96 (27,0) | 76 (30,3) |
| (65,100] | 20 (7,4) | 40 (10,1) | 39 (9,7) | 40 (10,0) | 34 (9,6) | 19 (7,6) |
| *Unknown* | *0* | *0* | *0* | *0* | *0* | *0* |

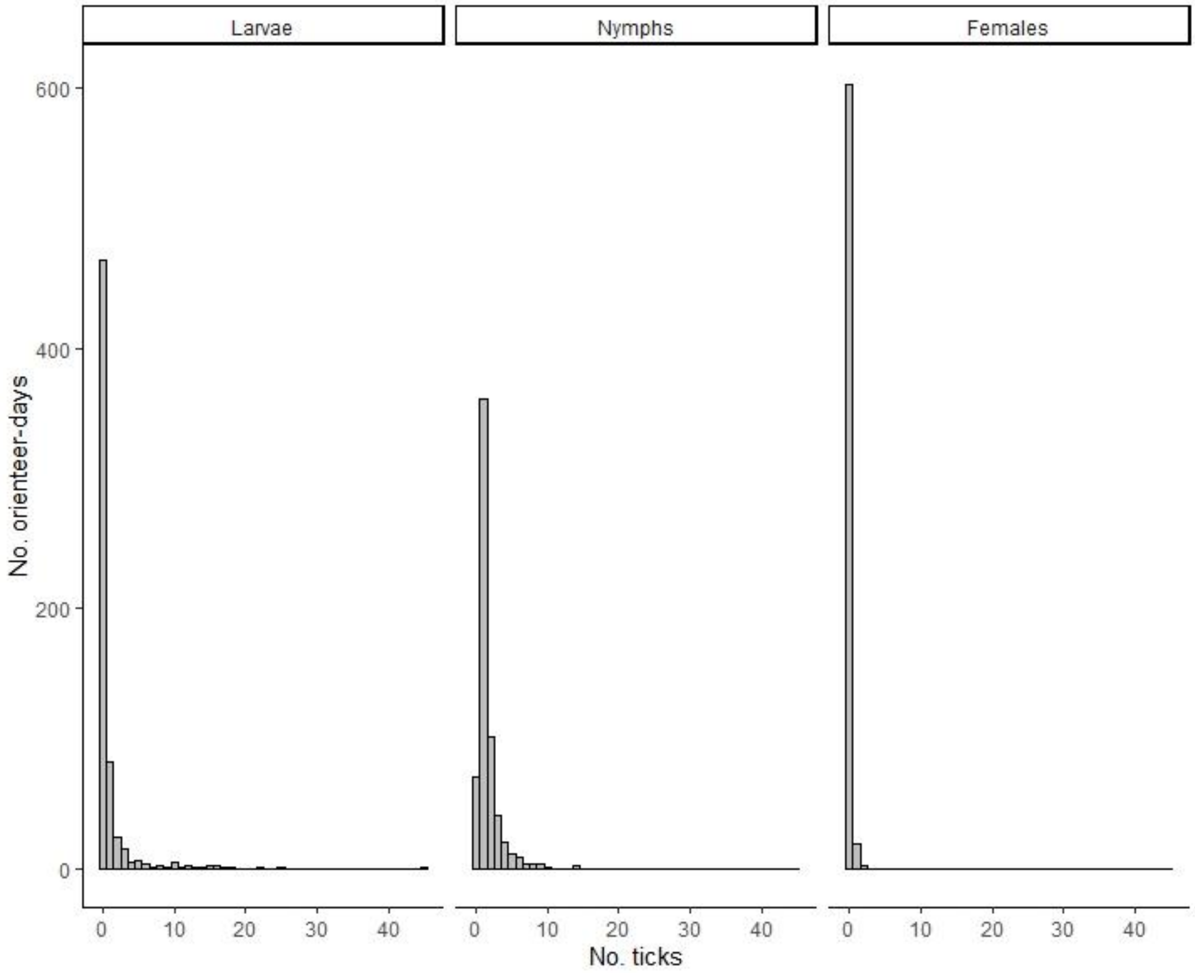


**Figure S1.** Histogram of the number of larvae, nymphs and females per orienteer-day having submitted ticks (n=626).
